# Antibiofilm effect enhanced by modification of 1,2,3-triazole and palladium nanoparticles on polysulfone membranes

**DOI:** 10.1038/srep24289

**Published:** 2016-04-12

**Authors:** Hong Cheng, Yihui Xie, Luis Francisco Villalobos, Liyan Song, Klaus-Viktor Peinemann, Suzana Nunes, Pei-Ying Hong

**Affiliations:** 1 Chongqing Institute of Green and Intelligent Technology, Chinese Academy of Sciences, Chongqing 401122, China; 2 King Abdullah University of Science and Technology (KAUST), Water Desalination and Reuse Center (WDRC), Biological and Environmental Sciences & Engineering Division (BESE), Thuwal, 23955-6900, Saudi Arabia; 3 King Abdullah University of Science and Technology (KAUST), Biological and Environmental Sciences & Engineering Division (BESE), Thuwal, 23955-6900, Saudi Arabia; 4 King Abdullah University of Science and Technology (KAUST), Advanced Membrane and Porous Materials (AMPM), Physical Sciences & Engineering Division (PSE), Thuwal, 23955-6900, Saudi Arabia

## Abstract

Biofouling impedes the performance of membrane bioreactors. In this study, we investigated the antifouling effects of polysulfone membranes that were modified by 1,2,3-triazole and palladium (Pd) nanoparticles. The modified membranes were evaluated for antibacterial and antifouling efficacy in a monoculture species biofilm (i.e., drip flow biofilm reactor, DFR) and mixed species biofilm experiment (i.e., aerobic membrane reactor, AeMBR). 1,2,3-triazole and Pd nanoparticles inhibited growth of *Pseudomonas aeruginosa* in both aerobic and anaerobic conditions. The decrease in bacterial growth was observed along with a decrease in the amount of total polysaccharide within the monoculture species biofilm matrix. When the modified membranes were connected to AeMBR, the increase in transmembrane pressure was lower than that of the non-modified membranes. This was accompanied by a decrease in protein and polysaccharide concentrations within the mixed species biofilm matrix. Biomass amount in the biofilm layer was also lower in the presence of modified membranes, and there was no detrimental effect on the performance of the reactor as evaluated from the nutrient removal rates. 16S rRNA analysis further attributed the delay in membrane fouling to the decrease in relative abundance of selected bacterial groups. These observations collectively point to a lower fouling occurrence achieved by the modified membranes.

Membrane bioreactors (MBR) are increasingly used as a preferred biotechnology for wastewater treatment because the coupling of a membrane separation process would achieve an improved effluent quality. Membranes can also be used to retain catalytic metals (e.g. manganese oxide, palladium) within the bioreactors. The catalytic metals in turn achieve reductive hydrodehalogenation of contaminants (e.g. pharmaceutical and personal care products, biocides and organic micropollutants) that are otherwise not easily biodegraded in a conventional activated sludge process. To demonstrate, palladium (Pd) has been used as a catalytic metal to achieve reductive removal of pharmaceuticals, biocides and iodinated contrast media^1^. Similarly, Pd was used in a continuous plate membrane reactor to achieve a complete, efficient and rapid removal of trichloroethene (TCE)^2^. In both instances, Pd was either physically retained in the MBR through the use of hollow-fiber nanofiltration membranes or recirculated throughout the reactor system in the form of suspension. However, the lack of a support material for these nanoparticles can result in the agglomeration and growth of nanoparticles, which will cause a subsequent decrease in the catalytic effect^3^,^4^.

Alternatively, Pd particles can be embedded onto membrane surfaces so as to achieve an even distribution of this catalyst throughout the reactive surface area. For example, Hennebel and coworkers encapsulated Pd particles in polyvinylidene fluoride membranes and demonstrated that such modified membrane contactors can be used in the treatment of diatrizoate-contaminated water^5^. However, there exists a possibility that Pd-containing membranes would be susceptible to biofoulants’ deposition over the course of reactor operation. This is because unlike some heavy metals such as silver or copper nanoparticles showing strong antibacterial effects^6^,^7^,^8^,^9^, Pd nanoparticles only have weak and selective antibacterial effects^10^. Biofouling is particularly detrimental to the performance of membrane bioreactors as they can result in decreased permeate production, increased transmembrane pressure and a shorter lifetime of membrane module^11^. In addition, biofoulants like proteins and polysaccharides can poison the catalyst surface and result in low catalytic activity.

A strategy to overcome this limitation would be to incorporate functionally-active chemical groups into the Pd-encapsulated membranes. One example of such functionally-active chemical group is the triazole ligands. Triazole ligands had been found to exhibit cytotoxic effects on cancer cells^12^. It can also interact with cytochrome P450 which in turn inhibit biosynthesis of ergosterol in fungi. The end result is a detrimental impact on the membrane-bound enzymatic activities and membrane permeability for the fungal cells^13^,^14^. Furthermore, triazole ligands showed antibacterial effects by inhibiting the biosynthesis of C-55 isoprenoid alcohol and vitamin K. The deficiency of C-55 isoprenoid alcohol would in turn affect the biosynthesis of bacterial cell wall and membrane polymer; and the lack of vitamin K would have a detrimental impact in the electron transport of gram-positive bacteria^15^.

Till date, there are limited studies to demonstrate the use of triazole ligands as an antifouling agent in polymeric membranes. An earlier study had demonstrated that triazole-containing polymer had an inhibitory effect on the number of suspended and attached *Pseudomonas aeruginosa* cells recovered from a polymeric membrane^16^. Although it is inferred that an antibacterial effect would relate to a delay in the occurrence of membrane fouling, the study did not demonstrate the use of antibacterial membranes in an operational membrane bioreactor to achieve delayed membrane fouling. Neither did the study evaluate how triazole ligands achieve antifouling effect when functionalized onto membranes, or whether a synergistic antifouling effect can be achieved in the presence of both triazole and Pd.

In this study, we propose the embedment of Pd on PSU membrane functionalized with 1,2,3-triazole to minimize membrane fouling in both bacterial monoculture and complex biofilm experiments. The study first evaluates different concentrations of triazole, namely 23%, 49% and 94%, that were functionalized on the membranes. These membranes are subsequently referred to as PSU-TriN-23%, PSU-TriN-49% and PSU-TriN-94%. Second, palladium ions or palladium nanoparticles were embedded on PSU membranes functionalized with 94% triazole (i.e., PSU-TriN-Ions or PSU-TriN-NPs, respectively). The impacts these modified membranes had on protein, polysaccharide concentrations and on the number of intact bacterial cells in attached biofilm matrix were examined and compared against the respective control. Furthermore, the change in bioactivity level as a result of microbial community perturbation was characterized by 16S rRNA gene-based amplicon next-generation sequencing. This study aims to demonstrate that a PSU membrane modified with both triazole and Pd would show high antifouling properties, and to provide a multifaceted approach in studying how delay in membrane fouling would occur for such modified membranes.

## Results

### PSU membranes modified with triazole and palladium nanoparticles exhibit higher hydrophilicity and surface roughness

Triazole modification of the PSU membranes increased hydrophilicity of the PSU membranes except when PSU-TriN-94% were chelated with the Pd ions (Table 1). The PSU-TriN-NPs membrane showed the highest hydrophilicity (47.7°) compared to all other tested membranes, which ranged from 58.6° to 84.3°. Among all tested membranes, the PSU-TriN-NPs membrane had the roughest surface topography (R_a_ = 58.9). Both PSU-TriN-Ions and PSU-TriN-NPs membranes had about three-fold higher roughness arithmetic averages (R_a_) than PSU-TriN-23%, PSU-TriN-49%, PSU-TriN-94% membranes, and up to 9-fold higher surface roughness than PSU-Tri-0% membrane (Table 1, Figure S1).

### PSU membranes modified with triazole and palladium exhibit antibacterial effect against *Pseudomonas aeruginosa* PAO1

**Figure 1:**
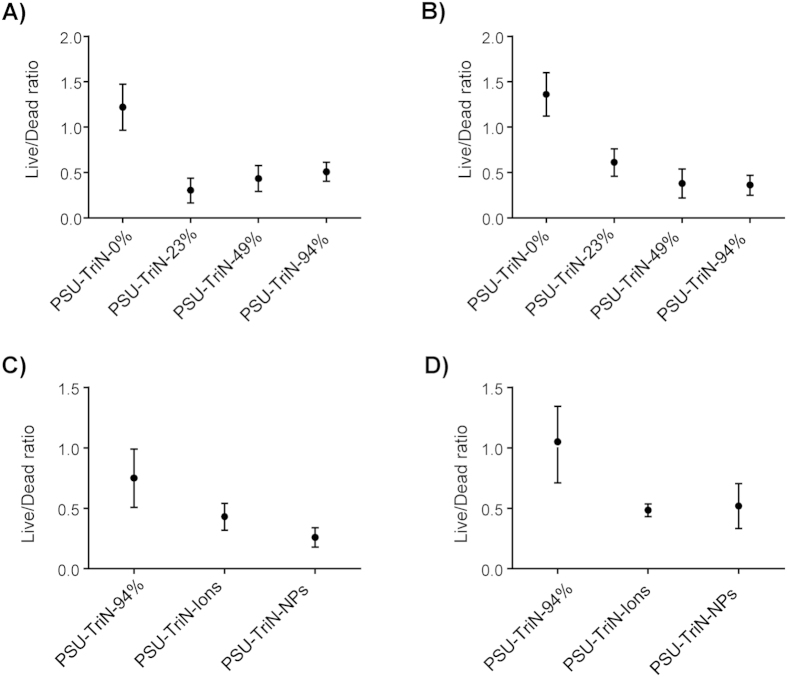
Live/dead ratio of bacteria attached on different membranes. (**A**) Different concentrations of triazole polysulfone membranes were tested in aerobic condition. (**B**) Four different concentrations of triazole polysulfone membrane in anaerobic condition. (**C**) PSU-TriN-94%, PSU-TriN-Ions, and PSU-TriN-NPs membranes were quantified in aerobic condition. (**D**) PSU-TriN-94%, PSU-TriN-Ions and PSU-TriN-NPs membranes in anaerobic condition.

The live/dead ratio of microorganisms strongly bound in the biofilm matrix of PSU-TriN-0%, PSU-TriN-23%, PSU-TriN-49% and PSU-TriN-94% were examined by confocal microscope (Figure S2). The live/dead ratio of biofilm matrix on PSU-TriN-0% was higher than 1 in both aerobic and anaerobic conditions. In contrast, the ratio of PSU-TriN-23%, PSU-TriN-49%, PSU-TriN-94% were less than 1, and was respectively found to be significantly lower than PSU-TriN-0%, which was 1.24 in aerobic and 1.36 in anaerobic conditions (t-test, P < 0.01) (Fig. 1A,B). Similarly, the number of intact cells in the biofilm matrix of PSU-TriN-0% was enumerated by flow cytometry, and was found to be significantly higher compared to triazole-functionalized PSU membranes in both aerobic and anaerobic conditions (t-test, all P < 0.05) (Figure S3A,B). Specifically, live cells in the biofilm of PSU membranes (PSU-TriN-0%) were about 5-fold higher in aerobic and approximately 8-fold higher in anaerobic conditions compared to the remaining three membranes. In addition, the tightly-bound biofilm, which is represented as the difference between total biofilm and loosely-bound biofilm (Figure S3A,B) had up to 1.8 × 10^7^ cells/(mL · cm^2^) and 3.95 × 10^8^ cells/(mL · cm^2^) on PSU-TriN-0% in both aerobic and anaerobic conditions, respectively. The number of intact cells was higher than that observed on the PSU-TriN-23%, PSU-TriN-49% and PSU-TriN-94% membranes.

The live/dead ratio on PSU-TriN-NPs were 0.26 in aerobic condition and 0.52 in anaerobic condition, indicating the presence of Pd nanoparticles can decrease live/dead ratio significantly in aerobic (t-test, P = 0.00) and anaerobic conditions (t-test, P = 0.03) compared to PSU-TriN-94% (ratio of 0.77 and 0.86, in aerobic and anaerobic condition, respectively) (Fig. 1C,D). The PSU-TriN-Ions membrane could also further reduce the live/dead ratio to 0.43 and 0.48 in aerobic and anaerobic condition, respectively, when compared to PSU-TriN-94% membrane (t-test, P < 0.05) (Fig. 1C,D).

### PSU membranes modified with triazole and palladium nanoparticles significantly reduced polysaccharides but not protein production from *Pseudomonas aeruginosa* PAO1

**Figure 2:**
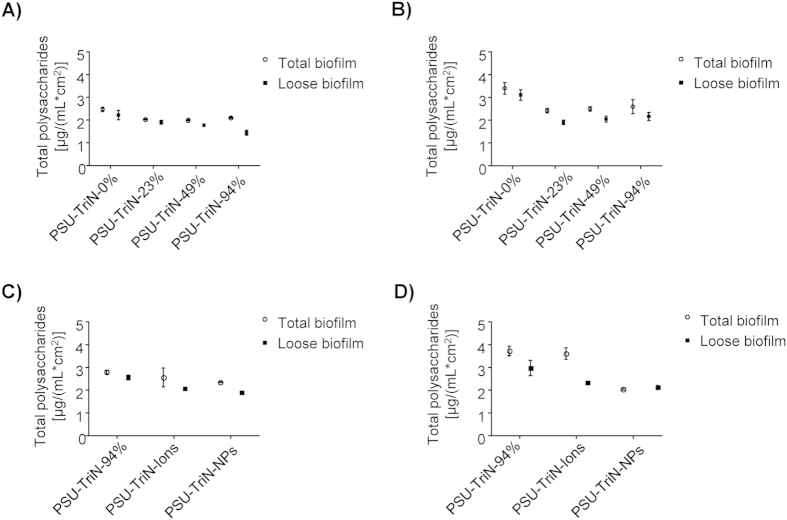
Quantification of total polysaccharides in the biofilm attached on (**A**) different concentrations of triazole membranes in aerobic condition, (**B**) different concentrations of triazole membranes in anaerobic condition, (**C**) PSU-TriN-94%, PSU-TriN-Ions, and PSU-TriN-NPs in aerobic condition, (**D**) PSU-TriN-94%, PSU-TriN-Ions and PSU-TriN-NPs in anaerobic condition.

In both aerobic and anaerobic conditions, *P. aeruginosa* PAO1 produced 2.34 ± 0.20 μg/(mL · cm^2^) total polysaccharides in aerobic condition and 3.25 ± 0.26 μg/(mL · cm^2^) in anaerobic condition within its biofilm matrix on PSU-TriN-0% membranes. The amount of polysaccharide on PSU-TriN-0% for both conditions were significantly higher than that on triazole-modified membranes. (i.e., PSU-TriN-23%, PSU-TriN-49%, PSU-TriN-94%) (t-test, all P < 0.03) (Fig. 2A,B). A significant difference could also be obtained between PSU-TriN-94% and PSU-TriN-NPs membranes (Fig. 2C,D, t-test, P = 0.01 and 0.02, respectively, in aerobic and anaerobic conditions). However, there was no significant difference between PSU-TriN-94% and PSU-TriN-Ions membranes (t-test, P = 0.12 and 0.13, respectively, in aerobic and anaerobic conditions). Specifically, the lower polysaccharide production in the monoculture biofilm was due to the significantly lower concentration of exopolysaccharides (including Pel, Psl and alginate polysaccharides) produced by *P. aeruginosa* PAO1^17^ on the three triazole-modified membranes than PSU-TriN-0% membranes in both aerobic (t-test, P < 0.03) and anaerobic conditions (t-test, P < 0.02) (Figure S4A,B). A further significant reduction in the exopolysaccharides was observed for PSU-TriN-NPs but not PSU-TriN-Ions when compared to PSU-TriN-94% in both aerobic (t-test, P = 0.00 and 0.07, respectively) and anaerobic (t-test, P = 0.01 and 0.84, respectively) conditions (Figure S4C,D).

**Figure 3:**
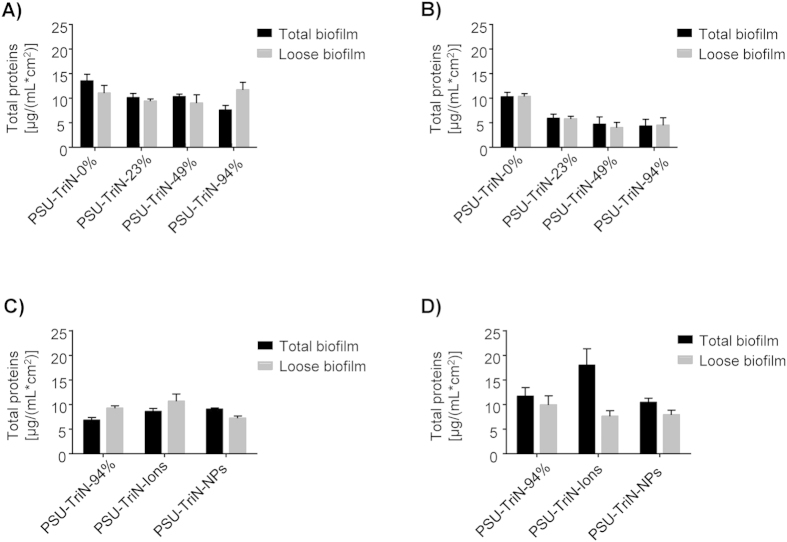
Proteins in biofilm established on PSU-TriN-0%, PSU-TriN-23%, PSU-TriN-49%, and PSU-TriN-94% in (**A**) aerobic and (**B**) anaerobic condition. Proteins in biofilm established on PSU-TriN-94%, PSU-TriN-Ions and PSU-TriN-NPs in (**C**) aerobic and (**D**) anaerobic conditions.

In contrast, the triazole-modified membranes only significantly reduced the protein production of *Pseudomonas aeruginosa* PAO1 in anaerobic conditions (t-test, all the P < 0.05) but did not show any significant reduction of proteins in aerobic conditions (Fig. 3A,B). A further comparison of PSU-TriN-94% membranes with PSU-TriN-Ions and PSU-TriN-NPs membranes also showed no significant difference for the expression of protein in both aerobic and anaerobic conditions (Fig. 3C,D).

### PSU-TriN-NPs showed delayed TMP increment when connected to aerobic membrane bioreactor

PSU-TriN-0%, PSU-TriN-94% and PSU-TriN-NPs were further evaluated for their antifouling effect by connecting them to a lab-scale aerobic membrane bioreactor (AeMBR) to establish mixed biofilm consortium. In both runs, PSU-TriN-NPs showed a lower TMP at the point of harvesting. To illustrate, both PSU-TriN-0% and PSU-TriN-94% membranes reached critical fouling on the 29th and 28th day of operation in run 1, respectively, but not PSU-TriN-NPs (Figure S5A). In run 2, the PSU-TriN-NPs only attained 10 kPa in its TMP which was lower than that for the remaining two membranes when the PSU-TriN-0% reached critical fouling on 21^th^ day (Figure S5B). All of the three membranes achieved an average chemical oxygen demand (COD) removal efficiency of 95% in both run 1 and run 2.

### Total polysaccharide concentration in soluble extracellular polymeric substance of biofilms were lower in the presence of PSU-TriN-NPs membranes

**Figure 4:**
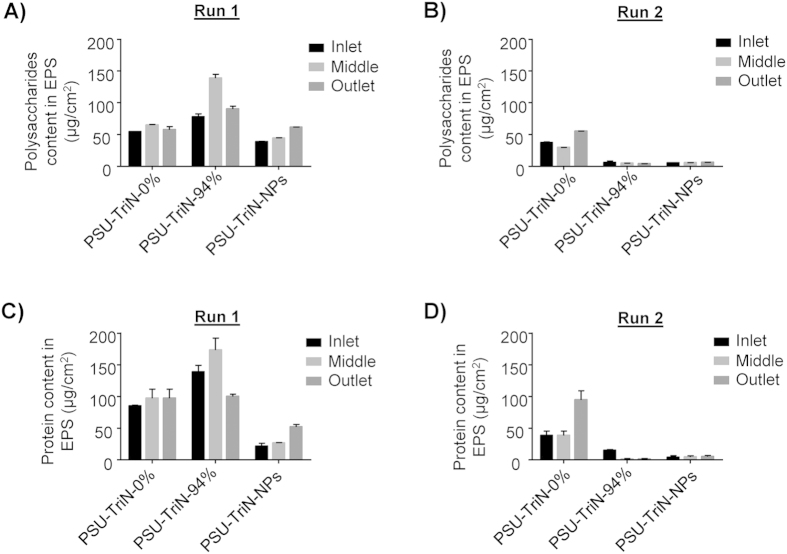
Polysaccharide and protein quantification in soluble extracellular polymeric substance (EPS) attached on PSU-TriN-0%, PSU-TriN-94% and PSU-TriN-NPs of run 1 and run 2. (**A**) Polysaccharide in run 1, (**B**) Polysaccharide in run 2, (**C**) Protein in run 1, (**D**) Protein in run 2.

The concentration of polysaccharide in the soluble EPS of biofilm attached onto PSU-TriN-NPs was 48.1 ± 10.7 μg/cm^2^. This concentration was significantly lower than the amount on the PSU-TriN-0% (59.0 ± 5.4 μg/cm^2^) and PSU-TriN-94% (102.3 ± 29.0 μg/cm^2^) membranes in run 1 (t-test, P = 0.05 and 0.00, respectively). In run 2, the concentration of polysaccharide on PSU-TriN-0% was 40.7 ± 12.0 μg/cm^2^. In contrast, there was a significant decrease to 5.86 ± 0.48 μg/cm^2^ of polysaccharide on PSU-TriN-NPs (t-test, P = 0.00) (Fig. 4A,B). In addition, quantification of polysaccharide in the retentate and permeate streams of AeMBR showed that the amount of retentate polysaccharide (11.4 μg/mL in run 1 and 8.0 μg/mL run 2) was higher than permeate (6.5 μg/mL, 6.5 μg/mL, 5.6 μg/mL in run 1 and 5.9 μg/mL, 2.7 μg/mL, 5.2 μg/mL in run 2, respectively, from PSU-TriN-0%, PSU-TriN-94%, PSU-TriN-NPs), indicating rejection of some polysaccharides by the membranes (Figure S6A,B).

### Total protein concentration in soluble extracellular polymeric substance of biofilms were lower in the presence of PSU-TriN-NPs membranes

The concentration of protein attached on PSU-TriN-NPs membrane was 34.14 μg/cm^2^ in run 1 and was significantly lower than the other two membranes (t-test, P = 0.00 and 0.00) (Fig. 4C). A similar result could be obtained for run 2. The concentration of protein attached on PSU-TriN-NPs membranes in run 2 was 4.40 μg/cm^2^, and was significantly lower than the tested membranes (t-test, P = 0.00) (Fig. 4D). Protein measurement was further verified by HPLC using a 280 nm detector. HPLC chromatograms showed a single-peak protein fragment of 0.93 kDa was observed in retentate and permeate streams of all three membranes (Figure S7), albeit at a higher concentration in run 1 compared to run 2. In contrast, the protein fragment profiles in the biofilm matrix of the three membranes were very different from that observed in the retentate and permeate streams. The protein fragment profiles on the membranes had molecular weight (MW) ranging from 0.15 kDa to 661.7 ± 40.2 kDa in run 1. Only the 661.7 kDa MW could be detected on the PSU-TriN-0% membrane in run 2 and the remaining palladium-modified membranes had no detectable fragments (Figure S7).

### PSU-TriN-NPs had lower ATP and AI-2 concentrations across membrane surface

**Figure 5:**
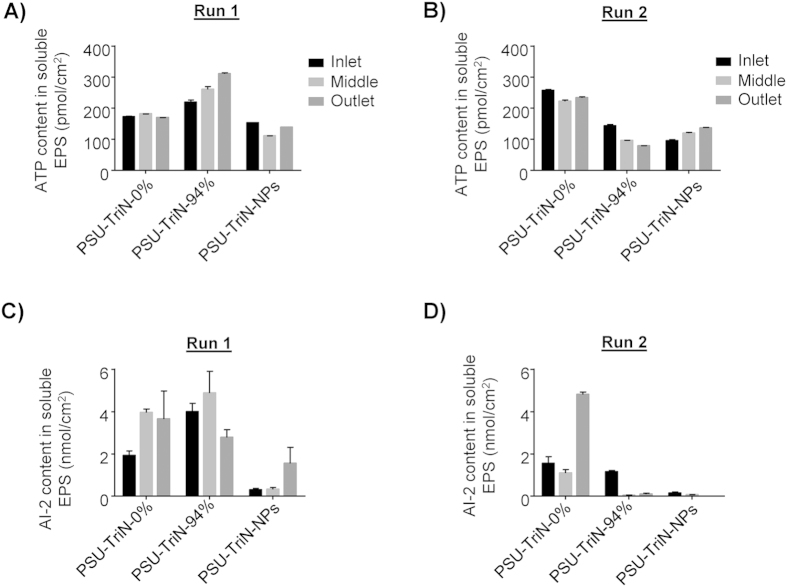
ATP and AI-2 measurement in EPS attached on PSU-TriN-0%, PSU-TriN-94 and PSU-TriN-NPs. (**A**) ATP concentration of run 1; (**B**) ATP concentration of run 2; (**C**) AI-2 amount of run 1; (**D**) AI-2 amount of run 2.

The average ATP concentration in the biofilm matrix attached on PSU-TriN-NPs membrane was about 133.59 ± 21.4 pmol/cm^2^ in run 1 and 116.60 ± 18.37 pmol/cm^2^ in run 2 (Fig. 5A,B). The presence of Pd nanoparticles on PSU-TriN membrane significantly decreased the ATP concentration in both run 1 and run 2 (t-test, P = 0.00). The average AI-2 concentration of PSU-TriN-0% and PSU-TriN-94% membranes were 3.18 and 3.88 nmol/cm^2^, respectively, and decreased to 0.72 nmol/cm^2^ on PSU-TriN-NPs membrane in run 1 (Fig. 5C). In run 2, the average AI-2 concentrations in PSU-TriN-0% membrane was 6 and 24 times higher than that detected in PSU-TriN-94% and PSU-TriN-NPs membranes, respectively (Fig. 5D).

### PSU-TriN-NPs affected the microbial community attached on the membrane

The presence of 94% concentration triazoles (i.e., PSU-TriN-94%) and Pd nanoparticles (i.e., PSU-TriN-NPs) on PSU-TriN-0% membrane resulted in a different microbial community (Figure S8, ANOSIM, R = 0.889 in run 1 and R = 0.815 in run 2, P = 0.1). To illustrate, the relative abundance of unclassified Acidobacteria Gp4, which is a predominant group detected in the biofilm matrix, accounted for 12.4% of total microbial community on PSU-TriN-NPs in run 1. This relative abundance was 35% lower than that on PSU-TriN-0%. In run 2, the same unclassified Acidobacteria Gp4 was also 52% lower in relative abundance on PSU-TriN-NPs than PSU-TriN-0%. Another predominant genus, *Acinetobacter,* was decreased by the presence of triazoles and NPs. The relative abundance was 6.2% on PSU-TriN-0%, which was 3.6-times and 2.4-times higher than that on PSU-TriN-NPs, respectively, in run 1 and run 2. Several OTUs that were identified to be affiliated with *Acidobacteria*, *Acinetobacter* and Chitinophagaceae exhibited a lower relative abundance when PSU-TriN-NPs membrane was used. For example, the abundance of *Blastocatella fastidiosa,* which is affiliated with the *Acidobacteria,* was present at a relative abundance of 10.0% on PSU-TriN-0% and was 1.5-times higher than that on PSU-TriN-NPs. Similarly, in run 2, its abundance in PSU-TriN-NPs decreased 1.9-times compared to PSU-TriN-0% (Table 2). Similar results can be obtained for other OTUs affiliated to *Acidobacteria* (e.g. *Blastocatella fastidiosa)*, *Acinetobacter* (e.g. *Acinetobacter guillouiae*) and Chitinophagaceae (e.g. *Terrimonas lutea*) (Table 2).

## Discussion

This study has systematically demonstrated that the functionalization of triazole ligands onto polymeric PSU membranes can result in antibacterial effects. The coupling of palladium nanoparticles on the same triazole-functionalized membrane (Figure S9) can further enhance the antibacterial effects, and delay the occurrence of membrane fouling when connected to on a lab-scale aerobic membrane bioreactor.

The use of heavy metals, albeit generally silver and copper but not palladium, on polymeric membranes to achieve antibacterial effect had been proposed in earlier studies^18^,^19^,^20^,^21^. For instance, Ag nanoparticles have been reported to show high biocide effects when functionalized onto forward osmosis membranes and block copolymer membranes^7^,^8^,^21^. Copper nanoparticles were also functionalized on a thin-film composite membrane to achieve antibacterial effects^18^. A similar proposition had also been made to use triazole ligands to inhibit the amount of intact cells attached onto membranes. For example, Duong and co-workers introduced the use of polytriazole on hydroxyl functionalized membranes, and showed the antibacterial and anti-attachment effects of such membranes^16^. Tejero *et al.* also showed that copolymers containing triazole functional groups exhibited high biocidal effects against many microorganisms (e.g. yeast*, Staphylococcus aureus, Pseudomonas aeruginosa*)^22^.

Unlike those studies which evaluated modified membranes for their antibacterial effects using monoculture bacterial model, this study provided a multifaceted approach to understand the mechanisms behind the antibacterial and antifouling effects of such modified membranes. First, triazole ligands can significantly decrease the live/dead ratio of bacteria attached on membrane (Fig. 1). Similarly, the ligands also effectively inhibited the growth of bacteria (Figure S3) in both loosely-bound portion and total of the biofilm in both aerobic and anaerobic conditions. This is in contrast to the unmodified PSU-TriN-0% membrane which had a relatively larger portion of the intact cells in the tightly-bound portion of the biofilm (Figure S3). This finding suggests that PSU-TriN-0% membrane was more prone to cell adhesion by bacteria, likely due to the relatively higher hydrophobicity of this membrane compared to those functionalized with triazoles^23^ (Table 1). However, there were no significant improvement in the extent of antibacterial activity arising from the different concentrations of triazoles functionalized onto the PSU membranes. This is exemplified from the absence of any significant reduction in total polysaccharides, live/dead ratio and live cells enumeration with increasing triazole concentrations, hence suggesting that the activity of triazole is not a limiting factor in achieving antibacterial effect. Instead, a combination with other materials, for example, palladium nanoparticles, are required to further enhance the antibacterial effect.

The inhibition of cell growth subsequently affected the production of bacterial polysaccharides in the biofilm matrix (Fig. 2A,B). Polysaccharides and proteins comprise as the main components of extracellular polymeric substance (EPS), which in turn account for 90% dry weight of a biofilm matrix^24^. The reduction of polysaccharides is hence likely to result in a delayed biofilm formation on the membrane. However, the inhibitory effect on proteins was not consistent in the monoculture and mixed species biofilm experiments. PSU-TriN-NPs can significantly decrease the amount of protein in mixed species biofilm matrix (Fig. 4C,D) while the presence of different concentration of triazole ligands did not result in any significant reduction of protein concentration compared to the control (Fig. 3A). Neither did the addition of palladium (i.e. ions, NPs) result in any significant reduction of the protein concentration in the monoculture species biofilm (Fig. 3C,D). Given that LB broth was used to establish the monoculture biofilm, the high concentration of protein content in this media may have contributed to background interference when using Lowry method to quantify for protein content in certain instances. Alternatively, because the method used (i.e., Lowry method) to quantify protein contents is not able to distinguish between proteins that are lysed out of dead bacterial cells and those associated with intact cell membranes, it is possible that the high protein content associated with the modified membranes were intracellular proteins lysed out from dead bacterial cells.

Instead, the protein content in AeMBR experiment showed significant difference between the modified membranes and PSU-TriN-0% (Fig. 4). Given the low protein concentration in SMP (Figure S6), there may be less interference on the Lowry method unlike the monoculture biofilm experiment. To verify, HPLC was used as an alternative method to detect and measure protein fragments in mixed species biofilm matrix. As the detection mechanism of HPLC is based on liquid chromatography, it is not subjected to the same limitation as the Lowry method. With this alternative method, it was shown that protein fragments measured on the modified membranes were barely detectable (Fig. 4 and Figure S7), hence indicating that membranes modified with triazole and Pd NPs could decrease the amount of proteins.

Besides affecting the production of polysaccharides and proteins, the inhibition of bacterial cell growth also correlated with a consequential decrease in the ATP and AI-2 concentrations per unit surface area, both of which can be used as parameters to indicate the amount of biomass. Further comparing the proteins in soluble EPS extracted from the biofilm on PSU-TriN-0%, it was observed that the molecular weight distributions of proteins were distinctly different from that detected in SMP (Figure S7). This suggests that certain protein portions in EPS were not from the direct absorption of proteins from SMP but rather, arise from the bacterial activity. Such activity was not apparent in the modified membranes as observed from the distinct lack of detectable protein fragments. The change in the bioactivity level was likely due to changes in the microbial communities on the modified membranes that are distinct from the control membrane (Figure S8). To illustrate, the relative abundance of unclassified Acidobacteria, unclassified Chitinophagaceae and *Acinetobacter* were lower in the presence of triazole and palladium. *Terrimonas lutea* was further identified at the OTU level to be consistently lower in the presence of the modified membranes compared to the control membrane (Table 2). Previous studies reported that family Chitinophagaceae was abundant in the biofilm of copper tube in drinking water distribution system^25^. Unclassified Chitinophagaceae including *Terrimonas rubra* was further reported to be involved in attachment on aerobic membrane, and that its abundance increased with the extent of membrane fouling^26^. Similarly, genus *Acinetobacter* and *Acidovorax* were reported to correlate to biofilm formation and to the abundance of quorum-sensing signal molecules^27^,^28^ (Table 2). The lower relative abundance of such bacterial groups by the modified membranes suggests a slower accumulation and formation of bacterial biofilm on these membranes.

Collectively, the decrease in the number of intact cells (i.e., viable cells), polysaccharide and protein content within the biofilm matrix attached on the membranes demonstrated the antibacterial effect of the modified membranes. These findings accounted for the slower TMP increment when the modified membranes were connected to aerobic MBR, suggesting that the membranes are less prone to biofouling. This is despite the addition of Pd ions and nanoparticles onto membranes that resulted in rougher surface topography (Figure S10, Table 1). It was previously demonstrated that membranes with rougher surface are more prone to fouling as roughness can increase the chance of cell-to-surface interaction and enhance adhesion of bacteria^23^,^29^. Regardless, our observations suggest that Pd nanoparticles, and to a lesser extent Pd ions, had antibacterial effects as the presence of Pd in combination with triazole, not only offset the influence of rougher topography but also enhanced the antifouling effects.

To conclude, the results in this study demonstrated that the 1,2,3-triazole modification can inhibit bacterial growth, and decrease the amount of polysaccharides and proteins. The triazole membrane embedded with Pd nanoparticles demonstrated synergistic effect in its antibacterial effects. When connected to an aerobic membrane bioreactor, the modified membranes were able to lower transmembrane pressure and delay the membrane biofouling process.

## Materials and Methods

### Synthesis of modified PSU membranes

Six types of PSU membranes were evaluated in this study. These included four different concentrations of triazole (i.e., 0%, 23%, 49% and 94%) functionalized onto PSU membranes, and referred to as PSU-TriN-0%, PSU-TriN-23%, PSU-TriN-49%, PSU-TriN-94% in this study. Owing to the fact that only PSU-TriN-94% membrane can embed the Pd ions and nanoparticles, PSU-TriN-94% membranes containing either palladium ions (PSU-TriN-Ions) or palladium nanoparticles (PSU-TriN-NPs) were evaluated in this study. PSU-TriN with different concentrations of triazole was synthesized based on a published method^30^. Briefly, polysulfone was chloromethylated in the presence of phenyl rings and 1,4-disubstituted 1,2,3-triazole rings were incorporated by copper(I)-catalyzed azide-alkyne cycloaddition. PSU-TriN-0%, PSU-TriN-23%, PSU-TriN-49%, and PSU-TriN-94% membranes were made by non-solvent (i.e. water) induced phase separation, and 20 wt% polymer solutions in N,N-dimethylacetamide (DMAc). In all syntheses, the polymer solution films as thick as 200 μm were casted over the polyester non-woven support. PSU-TriN-Ions and PSU-TriN-NPs membranes were made by complexation-induced phase separation^31^ using Pd(II) acetate (Pd(OAc)_2_) as the source of palladium. Briefly, a 200 μm film of polymer solution (20 wt% PSU-TrN-94% in DMAc) was cast over a non-woven support; and the resulting polymer solution film was immersed for 3 s in a 36 mM solution of Pd(OAc)_2_ in DMAc to form the palladium rich top layer. Finally, the polymer was immersed in a water bath to form the palladium-free porous support. At this point, the PSU-TriN-Ions membrane was obtained. An additional reduction step was performed to reduce the palladium ions to nanoparticles. This step consisted of immersing PSU-TriN-Ions for 5 min in a 0.05M solution of sodium tetrahydroborate (NaBH_4_), hence obtaining PSU-TriN-NPs membrane. PSU-TriN-NPs membranes synthesized in this manner had previously been found to be well-distributed on only the top layer of the polymeric membrane where the precursors (i.e., triazole) are^32^. The uniform layer of Pd nanoparticles on the membrane surface of PSU-TriN-NPs but not on the PSU-TriN-Ions membranes are further shown in Figures S10 and S11. All the membranes were kept in sterile deionized water before use.

### Membrane characterization

The FEI Nova Nano scanning electron microscope (SEM) and dark field Titan 80–300 CT transmission electron microscope (TEM) (Oregon, US) were used to observe the surface of membranes at 5 kV and 300 kV, respectively. The TEM microscope was also equipped with an X-ray energy dispersive spectroscopy (EDS) detector, a charge-coupled device camera and a post-column energy filter. To prepare sample for SEM, a piece of membrane sample was dried in air and then fixed onto a flat aluminum stub. 3 nm thick iridium was sputtered onto the membrane surface by a K575X Emitech sputter coater (Quorum Technologies, UK). To prepare the sample for TEM, the membranes were embedded in low-viscosity epoxy resin (Agar R1165) and cured at 60 °C for 24 h. Ultrathin sections (100 nm) were prepared with an ultramicrotome (Leica EM UC6) and put on a copper grid (180 mesh). Atomic force microscopy (AFM) was used to characterize the membrane surface topography. The air-dried membrane was fixed onto a glass slide. AFM imaging was performed by Bruker Dimension ICON equipment (Santa Barbara, CA, US) in a soft tap mode. For each membrane, a picture with 5 μm width and 5 μm length was scanned. Contact angle was measured to quantify the hydrophilicity of membranes by EasyDrop shape analyzer (Krüss, Hamburg, Germany) in static mode at ambient temperature. Ultra-pure water was used as the probing liquid and the mean values were determined from three different independent specimens.

### Reactor setup and operation

Drip-flow biofilm reactor (DFR) (Biosurface Technologies, Bozeman, MT, US) was used to establish monoculture biofilm on the membranes. The methods and conditions were as described previously^33^,^34^. Briefly, *P. aeruginosa* PAO1 was inoculated into 20 mL LB Broth (Lennox) (Sigma-Aldrich, St. Louis, MO, US), and the growth culture was incubated in a 200 rpm shaker incubator for 24 h at 37 °C. After that, the bacterial culture was diluted with LB broth to a 600 nm optical density (OD_600_) of 0.07. This OD measurement at 600 nm wavelength corresponds to an approximate cell density of 10^8^ cells/mL. The membranes were attached onto polycarbonate coupons, and tested in duplicates. The coupons were then placed into individual channels of autoclaved DFR, and incubated for 10 h at 37 °C. For anaerobic condition, 1% potassium nitrate w/v was added to LB broth to ensure the growth of *P. aeruginosa* PAO1 in the presence of nitrate as an alternative electron acceptor^35^. The membranes were harvested after 48 h of cultivation in the presence of a continuous 0.8 mL/min flow of LB broth in aerobic condition, and after 72 h of cultivation due to a slower growth rate under anaerobic condition. Two runs on the DFR in aerobic and two runs in anaerobic conditions were conducted.

An upflow-attached (UA) AeMBR with the same operating conditions as described earlier^26^ was also used in this study to establish mixed bacterial consortium on the membranes. Three external cross-flow flat sheet membrane modules (i.e., PSU-TriN-0%, PSU-TriN-94%, PSU-TriN-NPs) were connected to the reactor in series. Each membrane was operated with a constant trans-membrane flux of 6 to 8 L M^−2^ h^−1^ (LMH) under an average hydraulic retention time (HRT) of 18.5 h. All membranes were simultaneously harvested when any of the three membranes showed critical fouling. Soluble microbial products (SMP), including permeate from each membrane and retentate in the reactor, were sampled weekly throughout the operation based on protocol described earlier^26^. Two runs on the AeMBR were conducted to ensure reproducibility of results.

### Membranes harvesting and preparation procedure

For the DFR experiments, the duplicate membranes in each run were harvested and handled in slightly different manner. Briefly, one membrane was washed by 40 mL of 1X PBS to remove the loosely-bound biofilm and the membrane was subsequently kept for confocal microscopic analysis; and the other membrane was placed into 40 mL PBS without any prior washing step (i.e., both loosely- and strongly-bound biofilm were collected). The tubes containing the loosely-bound biofilm and the total biofilm were individually ultrasonicated for 3 min by a Q500 sonicator (Qsonica, Newton, CT, USA) at 25% amplitude with 2 s pulsating intervals to disperse biofilm into the liquid suspension. The membranes were then removed from the liquid suspension. A portion of the suspension was used for live-cell counting and Pel polysaccharide while the remaining was filtered through a 0.22 μm cellulose acetate membrane for polysaccharide and protein characterization.

For the AeMBR experiments, each harvested membrane with an area of 20 cm by 2.5 cm was aseptically cut into three equal parts. Each portion was named as inlet, mid and outlet, respectively, based on the direction of wastewater flow. Each portion was further subdivided for ATP quantification and soluble EPS measurements. To prepare for soluble EPS measurements, each membrane subportion was respectively immersed into 6 mL 1X PBS. The membranes were ultrasonicated in similar manner as described earlier. The membranes were then removed and the remaining suspensions were centrifuged at 9400 g for 20 min prior to filtration through 0.22 μm cellulose acetate. The cell pellets obtained after centrifugation were stored for DNA extraction and microbial community analysis.

### Live-cell counting of *P. aeruginosa* PAO1 monoculture biofilm

Two methods were used to enumerate live cells within the monoculture biofilm established on the PSU membranes. First, 1 mL of the liquid suspension was aliquot and diluted by 5000 to 7000-fold in 1X PBS for flow cytometry on Accuri C6 (BD Bioscience, NJ, US). LIVE/DEAD^®^ BacLight^TM^ Bacterial Viability and Counting Kit (Thermo Fisher Scientific, Waltham, MA, US) was used to stain the bacteria based on manufacturer’s protocol. Briefly, 300 μL of the 2X propidium iodide: Syto9 dye with respective concentrations of 30 μM and 6 μM was added to 300 μL of diluted liquid suspension and incubated in room temperature and in the dark for 15 min before flow cytometry. 50 μL stained samples were injected with 35 μL/min rate to enumerate cells with intact cell walls (i.e., live cells). Second, the membranes with the strongly-bound biofilm were stained by the LIVE/DEAD^®^ BacLight^TM^ Bacterial Viability and Counting Kit for 20 min. Zeiss LSM 710 (Carl Zeiss Microscopy GmbH, Jena, Germany) with 20X magnification objective was used. Image scanning was carried out with the 488 nm and 561 nm laser line from an Ar/Kr laser. 7 different images for each membrane were obtained, and the images were analyzed by Image J software^36^. During analysis, each image was split into two pictures according to the red and green channels of dead and live cells, respectively. Then the dead cells and live cells were individually counted to determine live/dead ratio was calculated.

### Polysaccharides measurement for *P. aeruginosa* PAO1 monoculture biofilm

*Pseudomonas aeruginosa* PAO1 is capable of producing Pel, Psl and alginate exopolysaccharides^17^. These exopolysaccharides are important in providing the structural scaffold of biofilm by *P. aeruginosa*^37^, and are anticipated to be the predominant polysaccharides within the monoculture biofilm matrix. The polysaccharides were measured by congo stain based on previous description with a slight modification. Briefly, prior to analysis, the liquid suspension was briefly vortex to ensure homogeneity in the contents. 10 mL of the homogeneous suspension was centrifuged at 9400 g for 10 min and discarded of their supernatant. The cell pellet was resuspended in 10 mL of 40 mg/mL congo red LB broth and incubated for 90 min in a 37 °C shaker incubator. After this, the suspension was centrifuged again at 9400 g for 10 min, and the supernatant was measured for its OD_490_. The total congo red absorbed in supernatant was measured. LB broth containing 5 mg/mL, 10 mg/mL, 20 mg/mL, 40 mg/mL, and 80 mg/mL congo red were also tested by OD_490_ to establish a standard curve for calculating congo concentration in supernatant.

### Total proteins (PN) and total polysaccharides (PS) determination

The supernatant was first filtered through 0.22 μm syringe filter (VWR US, Radnor, PA, US) prior to determination of its PN and PS. PN was quantified by Total Protein Kit, Micro Lowry, Peterson’s Modification (Sigma-Aldrich, St. Louis, MO, US) based on the Lowry method^38^, with 0 μg/mL, 10 μg/mL, 20 μg/mL, 40 μg/mL and 80 μg/mL of Bovine Serum Albumin (BSA) as standard and measured in triplicates. BSA originated from New Zealand and was made commercially available through Sigma-Aldrich in lyophilized powder under CAS number 9048-46-8. PS was determined by the phenol-sulfuric acid method. 0 μg/mL, 5 μg/mL, 10 μg/mL, 20 μg/mL, 40 μg/mL and 80 μg/mL glucose were used as standards^39^. Molecular weight (MW) analysis of proteins in SMP and EPS was conducted on a Water Breeze^TM^ 2 HPLC System (Waters Chromatography, Milford, MA, US) based on procedures described by Xiong *et al.*^26^.

### Adenosine Triphosphate (ATP) and Autoinducer-2 (AI-2) quantification

Adenosine triphosphate (ATP) and autoinducer-2 (AI-2) were quantified to measure the amount of biomass on the membranes. The membrane of dimensions 1 cm by 2.5 cm was placed into 2 mL deionized water, ultrasonicated for 2 min at 25% amplitude with 2 s pulsating intervals. ATP content in the suspension was quantified by the Celsis Amplified ATP ^TM^ reagent kit on an Advance luminometer (Celsis, Westminster, London, UK) with deionized water as a negative control. All the samples were measured in triplicates. The AI-2 from the liquid suspension of the harvested membranes was determined based on a previous protocol^26^.

### DNA extraction and 16S rRNA gene-based next generation sequencing

The genomic DNA of the pellets obtained from the soluble EPS extraction was extracted by the UltraClean^®^ Soil DNA Isolation Kit (MoBio Laboratories, Carlsbad, USA) with slight modifications^40^. PCR amplification of the 16S rRNA genes was applied with 515F (5′- Illumina overhang- GTGYCAGCMGCCGCGGTAA- 3′) and 907R (5′- Illumina overhang- CCCCGYCAATTCMTTTRAGT- 3′). All the amplicons were of the anticipated size of approximately 550 bp and the negative control has no amplification. PCR amplicons were cleaned up by AMPure XP beads (Beckman Coulter, CA, USA). After that, Index PCR was conducted to attach dual indices provided by the Nextera XT Index Kit (Illumina Inc, San Diego, CA, USA) based on the manufacturer’s protocol. Indexed PCR amplicons were cleaned up by AMPure XP beads (Beckman Coulter, CA, USA). Equimolar concentrations of the samples were mixed together and submitted to KAUST Core lab for Illumina MiSeq sequencing. The sequencing data were processed for its quality and analyzed by the same approach as specified in previous study^41^.

### Statistical analysis

The degree of similarities of the microbial communities attached onto the three kinds of membranes were analyzed by Primer E version 7^42^. Briefly, the abundances of the bacterial and archaeal genera were input into Primer E version 7, and square-root transformed before computing for their Bray-Curtis similarities. The Bray-Curtis similarities matrix was then used to construct a non-metric threshold multidimensional scaling (nMDS) plot by multivariate analysis. Bacterial targets that exhibited >0.7 correlation with the multivariate patterns on the nMDS were overlaid as vectors. ANOSIM analysis was used to determine if the observed clusters on nMDS were significantly different. All other significance tests were analyzed by two-tailed t-test on Microsoft Excel 2013 and Prism 6.

### Nucleotide sequence accession numbers

All high-throughput sequencing files were deposited in the Short Read Archive (SRA) of the European Nucleotide Archive (ENA) under study accession number PRJEB12586.

### Bioethics statement

All experimental protocols were carried out in accordance with the approved guidelines and were approved by Institutional Biosafety and Bioethics Committee, KAUST.

## Additional Information

**How to cite this article**: Cheng, H. *et al.* Antibiofilm effect enhanced by modification of 1,2,3-triazole and palladium nanoparticles on polysulfone membranes. *Sci. Rep.* **6**, 24289; doi: 10.1038/srep24289 (2016).

## Supplementary Material

Supplementary Information

## Figures and Tables

**Table 1:** Roughness parameters as determined by atomic force microscopy and hydrophilicity as evaluated by contact angle.

Membrane	Roughness			Hydrophilicity
	R_a_ (nm)^a^	R_q_ (nm)^b^	Image Z Range (nm)^c^	Contact angle(°)
PSU-TriN-0%	6.35	8.26	107	69.60 ± 0.75
PSU-TriN-23%	17.6	22.1	159	67.78 ± 0.91
PSU-TriN-49%	14.7	18.3	114	63.61 ± 0.52
PSU-TriN-94%	15.7	21.4	292	58.59 ± 0.79
PSU-TriN-Ions	56.8	76.3	543	84.31 ± 1.35
PSU-TriN-NPs	58.9	78.8	583	47.72 ± 1.34

^a^R_a_ is the arithmetic average of the absolute values of the surface height deviations measured from the mean plane.

^b^R_q_ is the root mean square average of height deviation taken from the mean image data plane.

^c^Z range is the distance of highest peak and lowest point.

**Table 2:** Average relative abundance of Operational Taxonomic Units (OTUs) changed with the types of membranes in run 1 and run 2.

	PSU-TriN-0% avg. (%)	PSU-TriN-94% avg. (%)	PSU-TriN-NPs avg. (%)	Identify
Run 1				
* Acidovorax delafieldii*	3.14	1.63	2.63	98%
* Blastocatella fastidiosa*	10.04	7.30	6.66	94%
* Lactococcus raffinolactis*	1.36	1.00	1.27	99%
* Aridibacter famidurans*	3.35	2.38	2.19	95%
* Terrimonas lutea*	0.36	0.35	0.35	98%
* Acinetobacter guillouiae*	4.89	1.21	1.36	98%
Run 2				
* Acidovorax delafieldii*	3.01	2.14	1.82	98%
* Blastocatella fastidiosa*	2.30	1.15	1.23	94%
* Lactococcus raffinolactis*	0.28	0.05	0.15	99%
* Aridibacter famidurans*	0.83	0.49	0.45	95%
* Terrimonas lutea*	0.96	0.45	0.67	98%
* Acinetobacter guillouiae*	0.21	0.10	0.09	98%
