# Supplementary material for: Antibiofilm effect enhanced by modification of 1,2,3-triazole and palladium nanoparticles on polysulfone membranes
Source: Sci Rep. 2016 Apr 12;6:24289. doi: 10.1038/srep24289 (PMC4828667; doi:10.1038/srep24289)

**Antibiofilm effect enhanced by modification of 1,2,3-triazole and palladium nanoparticles on polysulfone membranes**

Hong Cheng ^1,2^, Yihui Xie ^3^, Luis Francisco Villalobos ^4^, Liyan Song ^1^, Klaus-Viktor Peinemann ^4^, Suzana Nunes ^3^, Pei-Ying Hong *^2^

1. Chongqing Institute of Green and Intelligent Technology, Chinese Academy of Sciences, Chongqing 401122, China
2. King Abdullah University of Science and Technology (KAUST), Water Desalination and Reuse Center (WDRC), Biological and Environmental Sciences & Engineering

Division (BESE)

1. King Abdullah University of Science and Technology (KAUST), Biological and Environmental Sciences & Engineering Division (BESE)
2. King Abdullah University of Science and Technology (KAUST), Advanced Membrane and Porous Materials (AMPM), Physical Sciences & Engineering Division (PSE), Thuwal, 23955-6900, Saudi Arabia

* Corresponding author:

Pei-Ying Hong

Email: [peiying.hong@kaust.edu.sa](mailto:peiying.hong@kaust.edu.sa)

Phone: +966-12-8082218


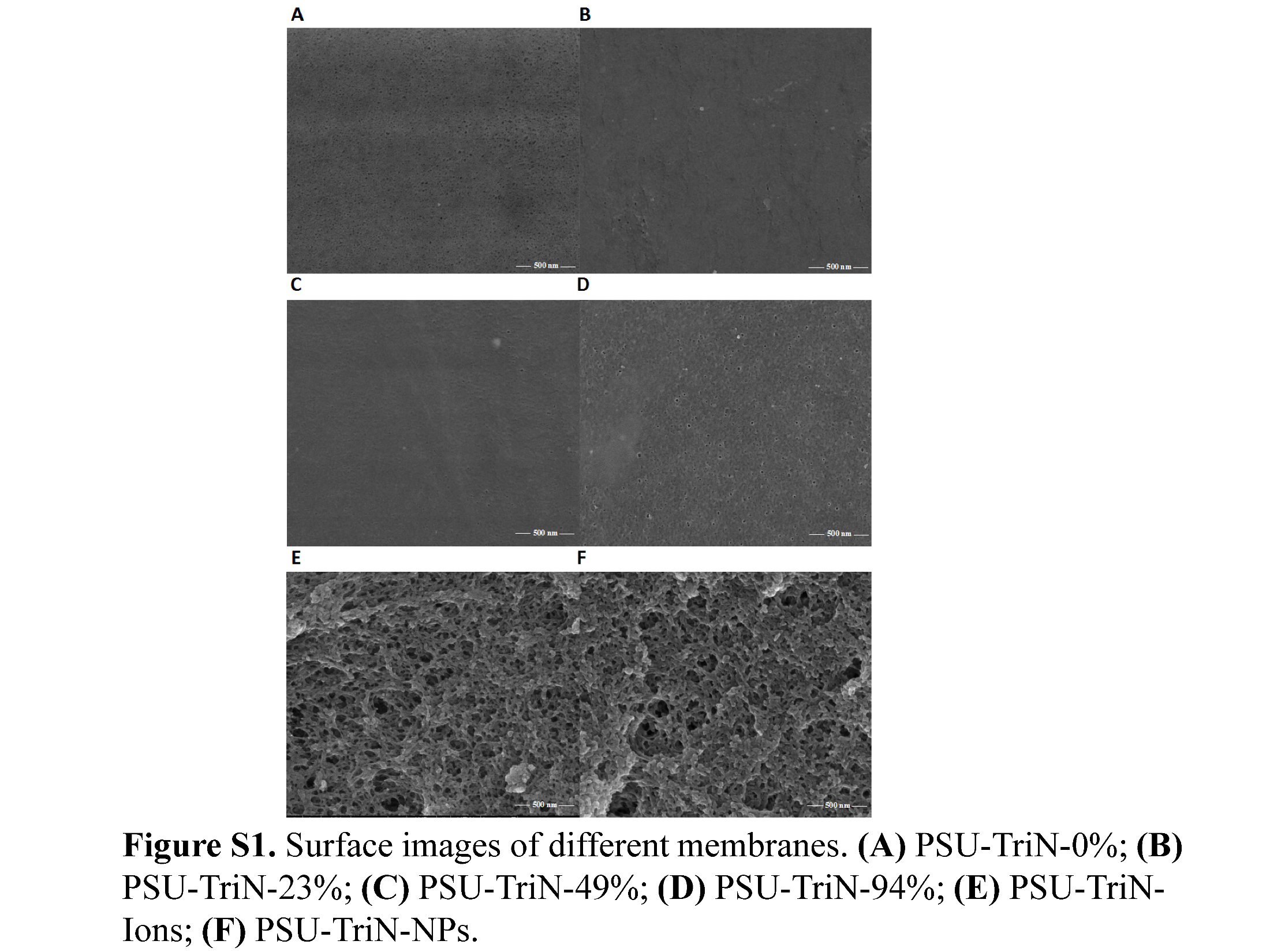


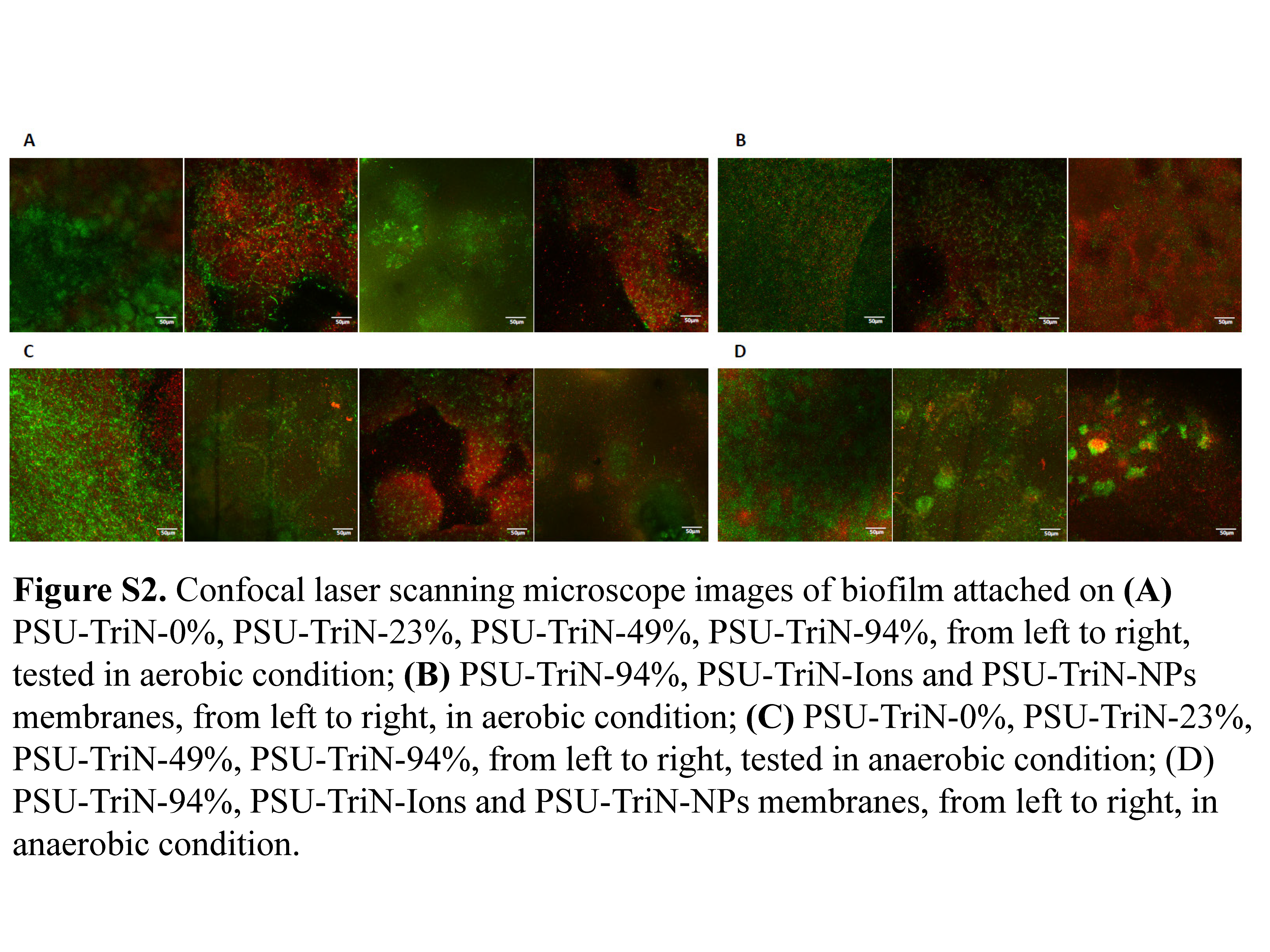


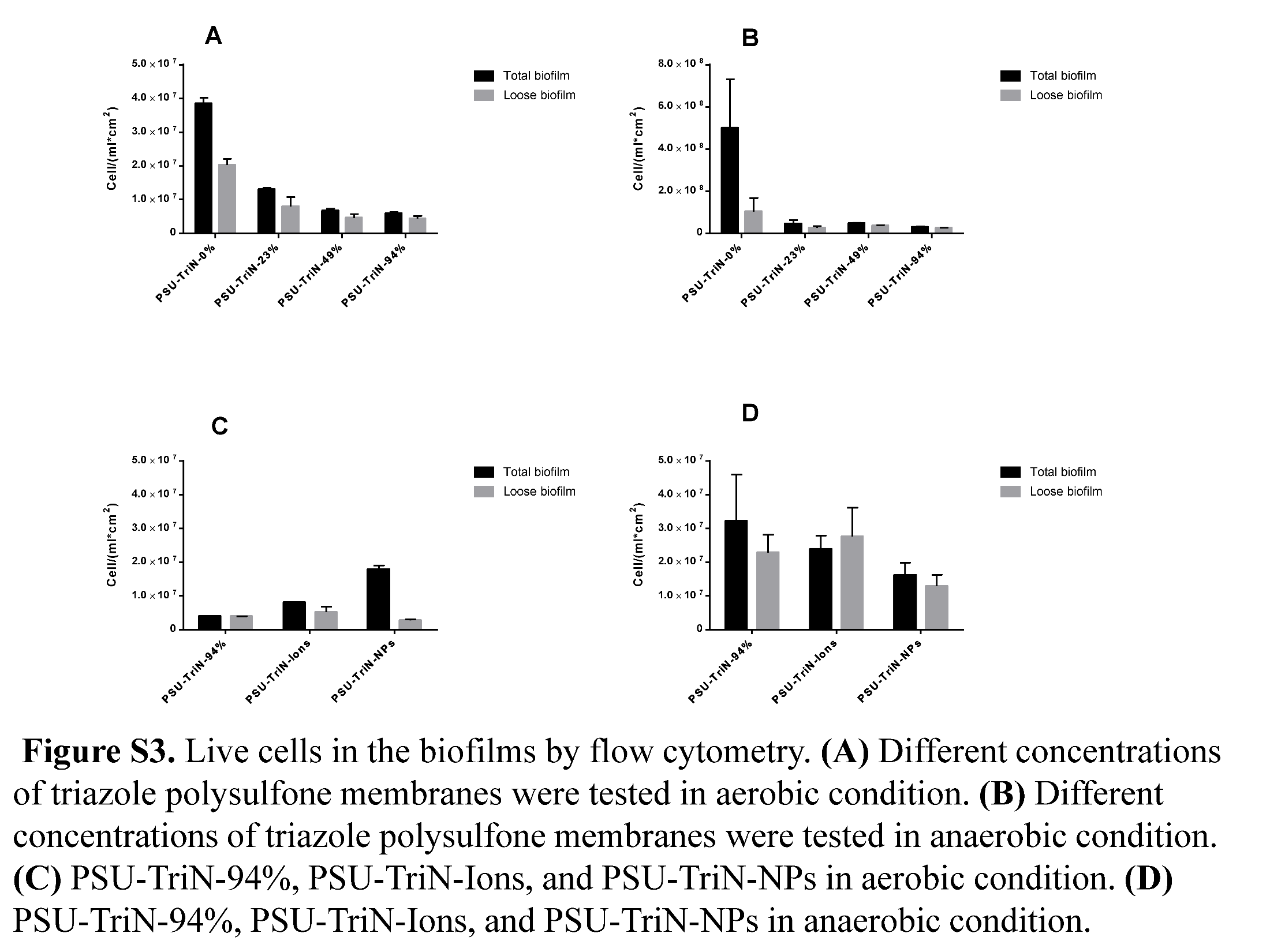


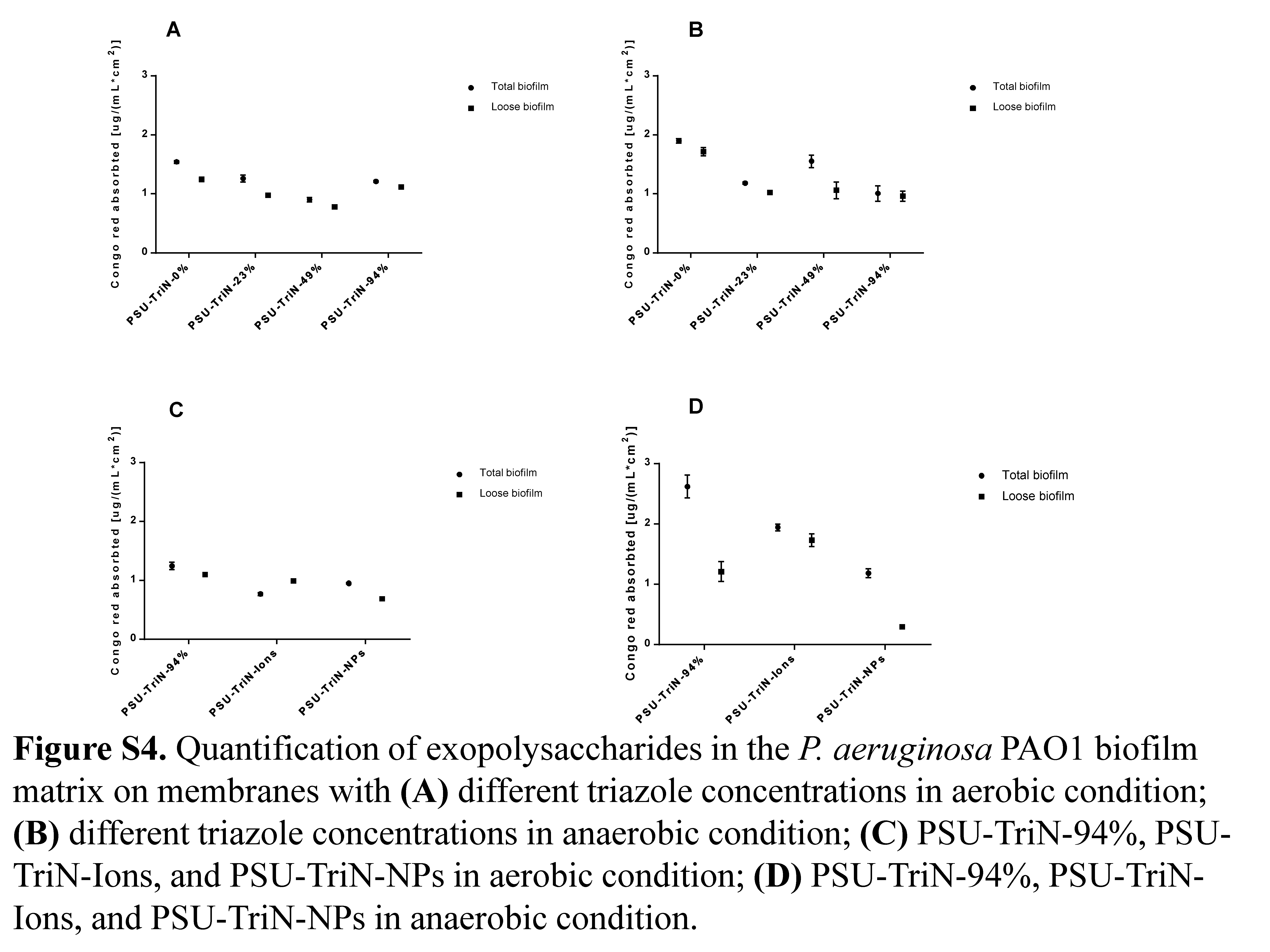


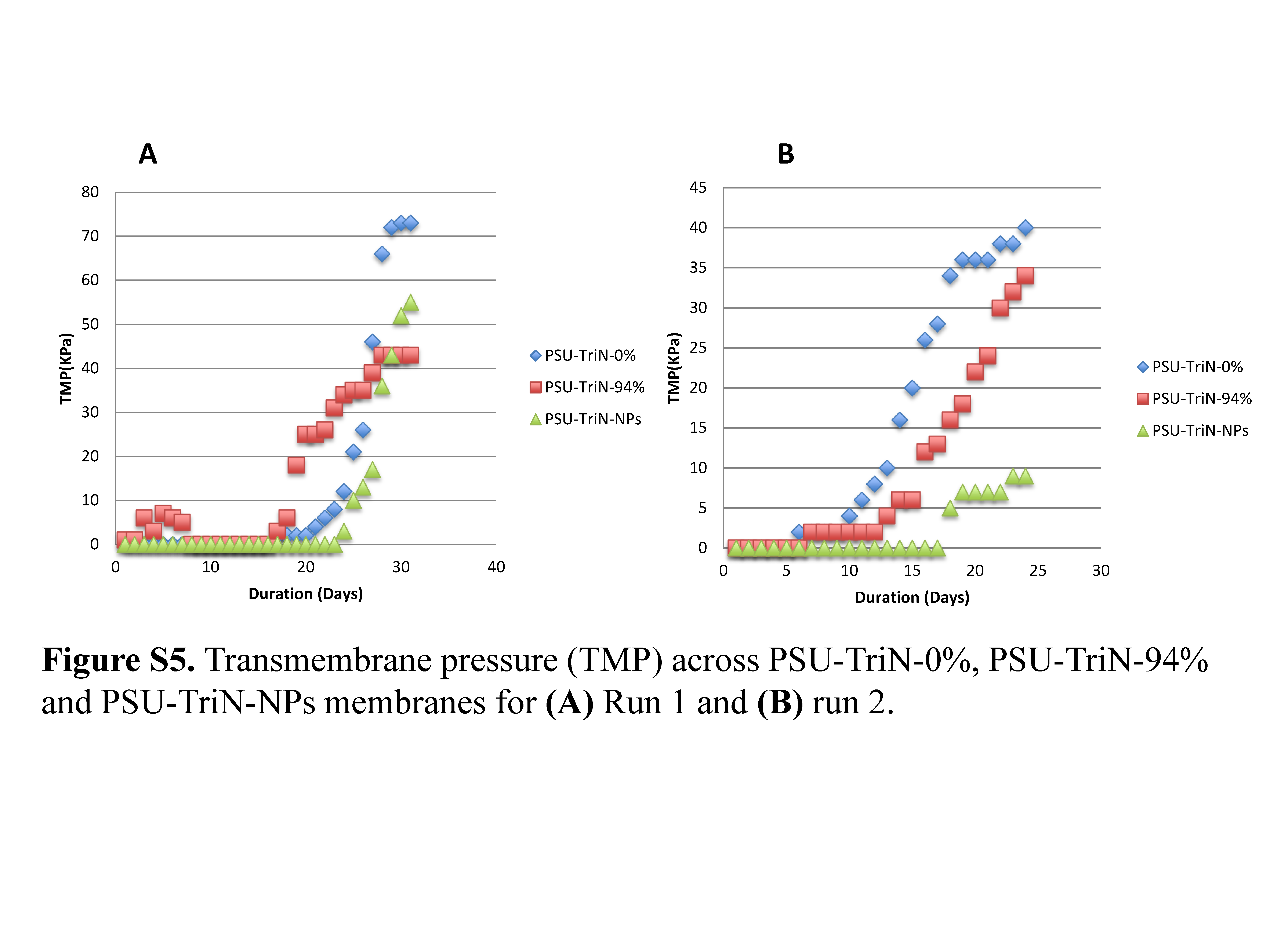


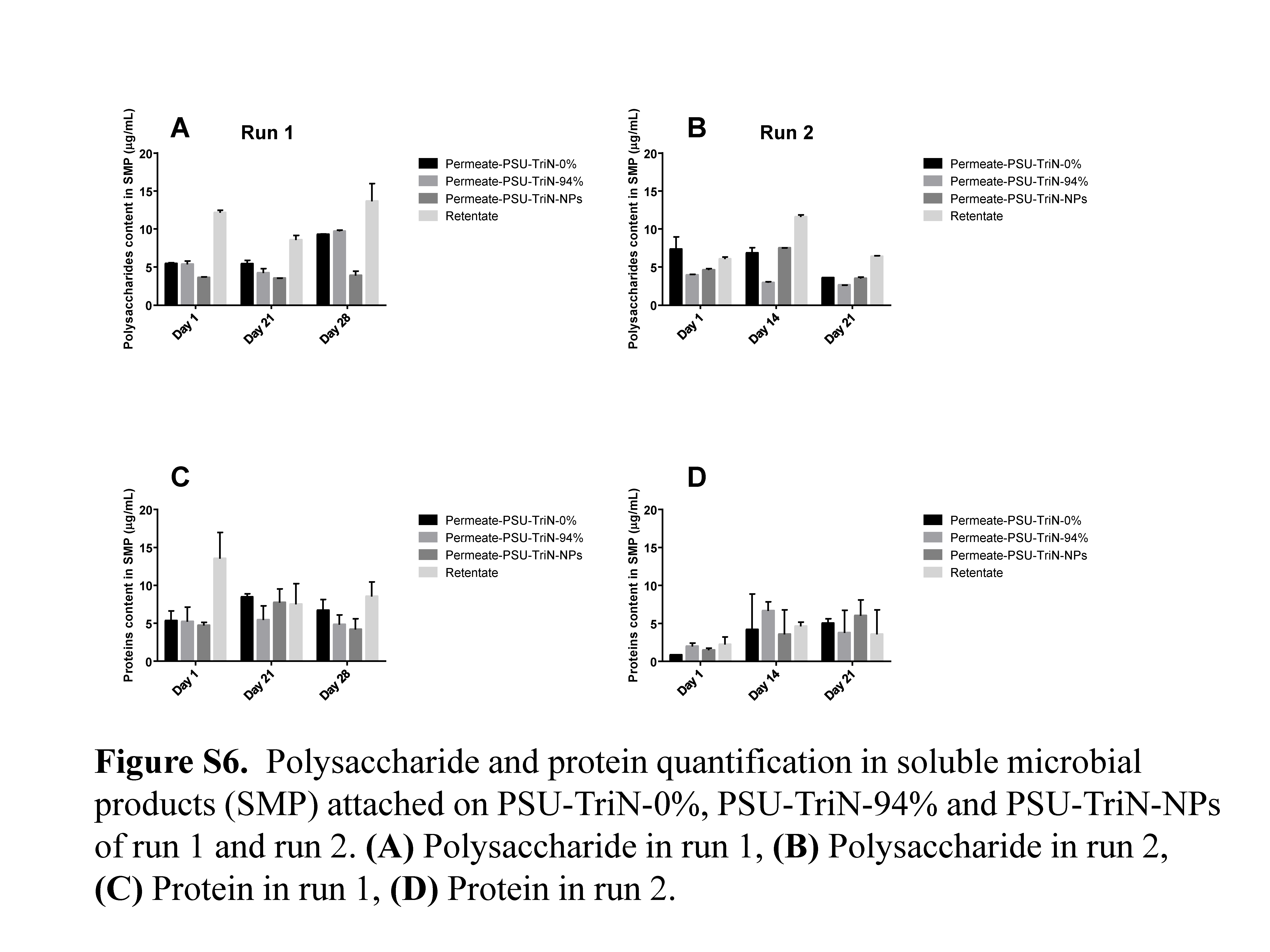


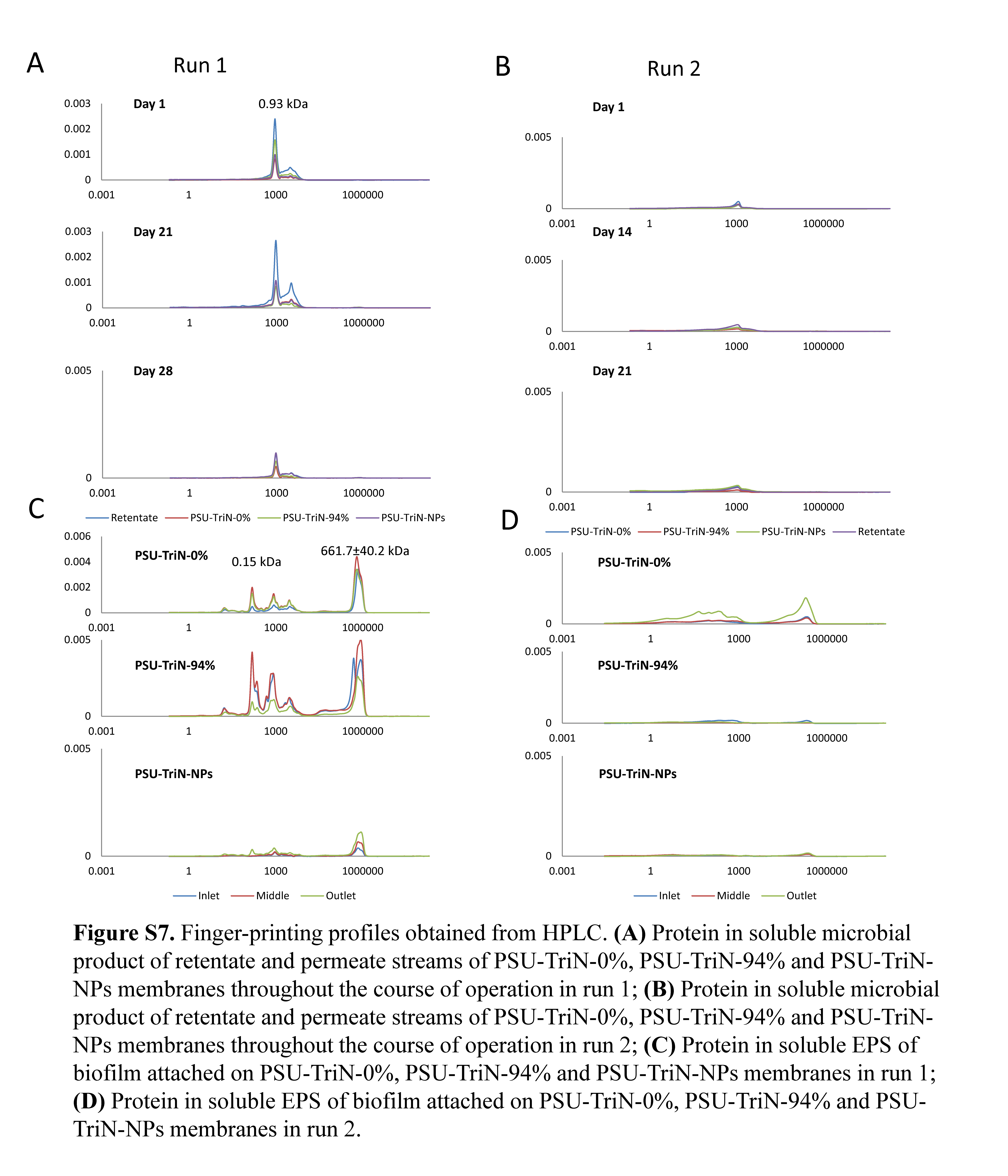


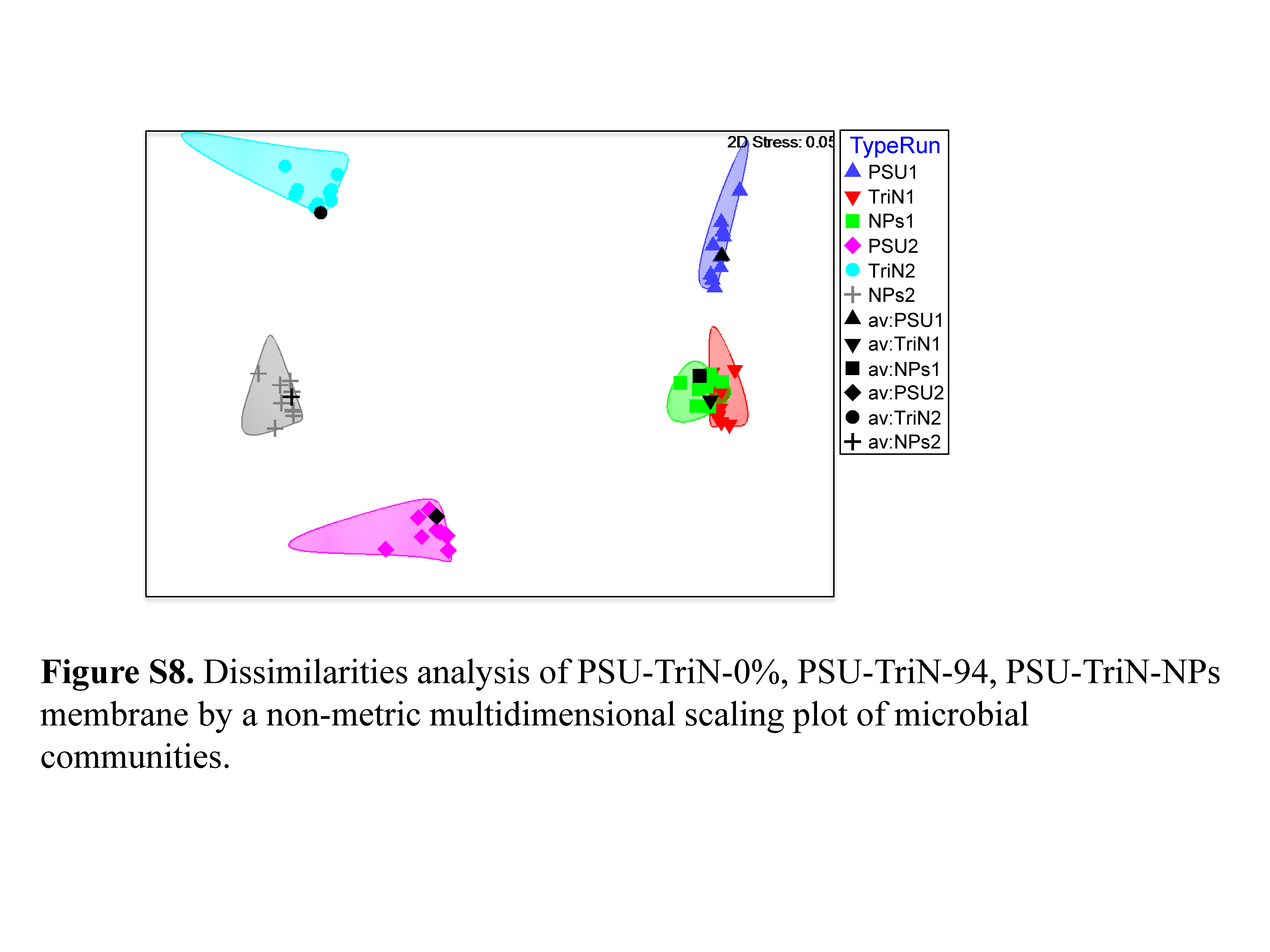


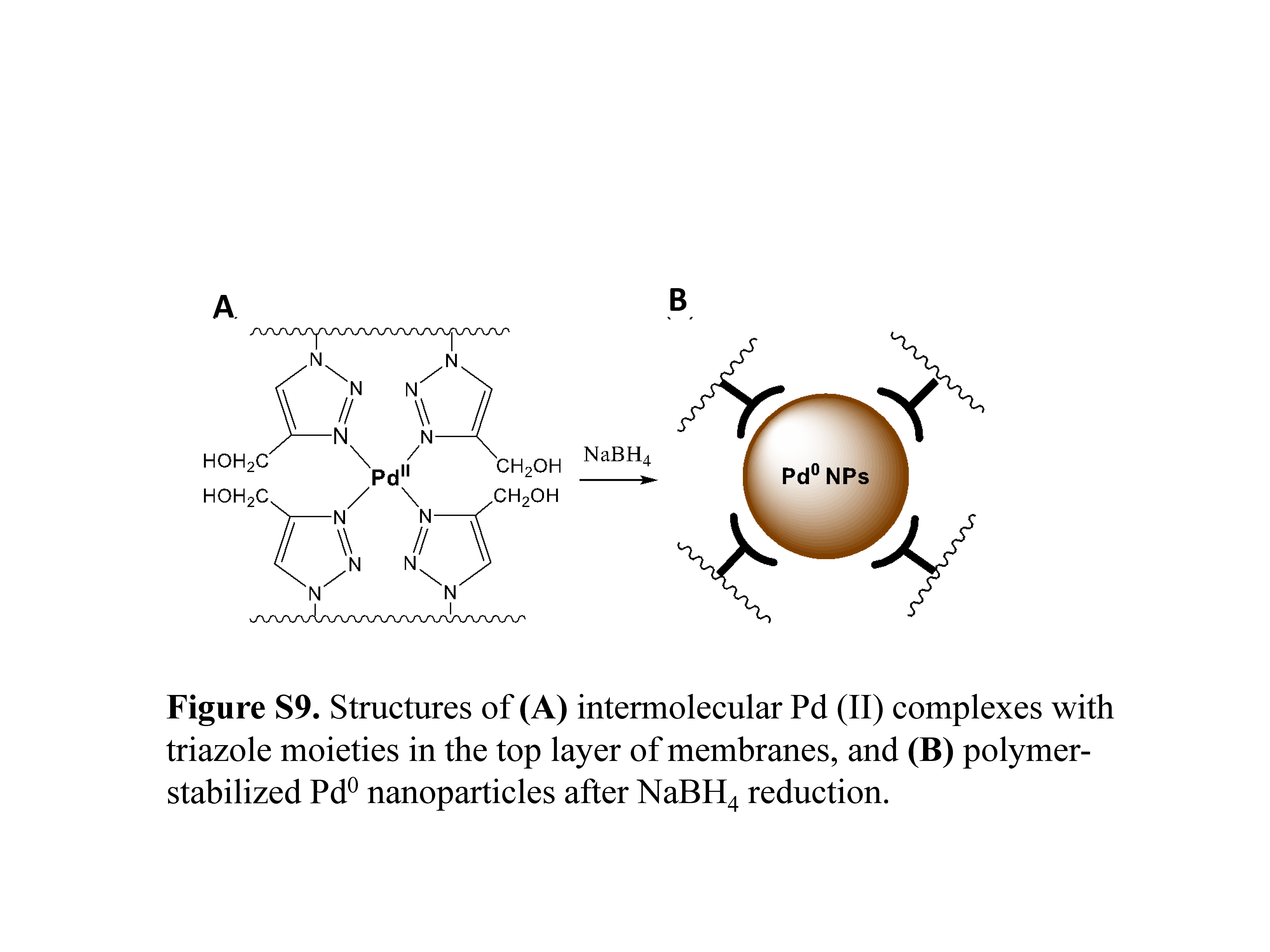


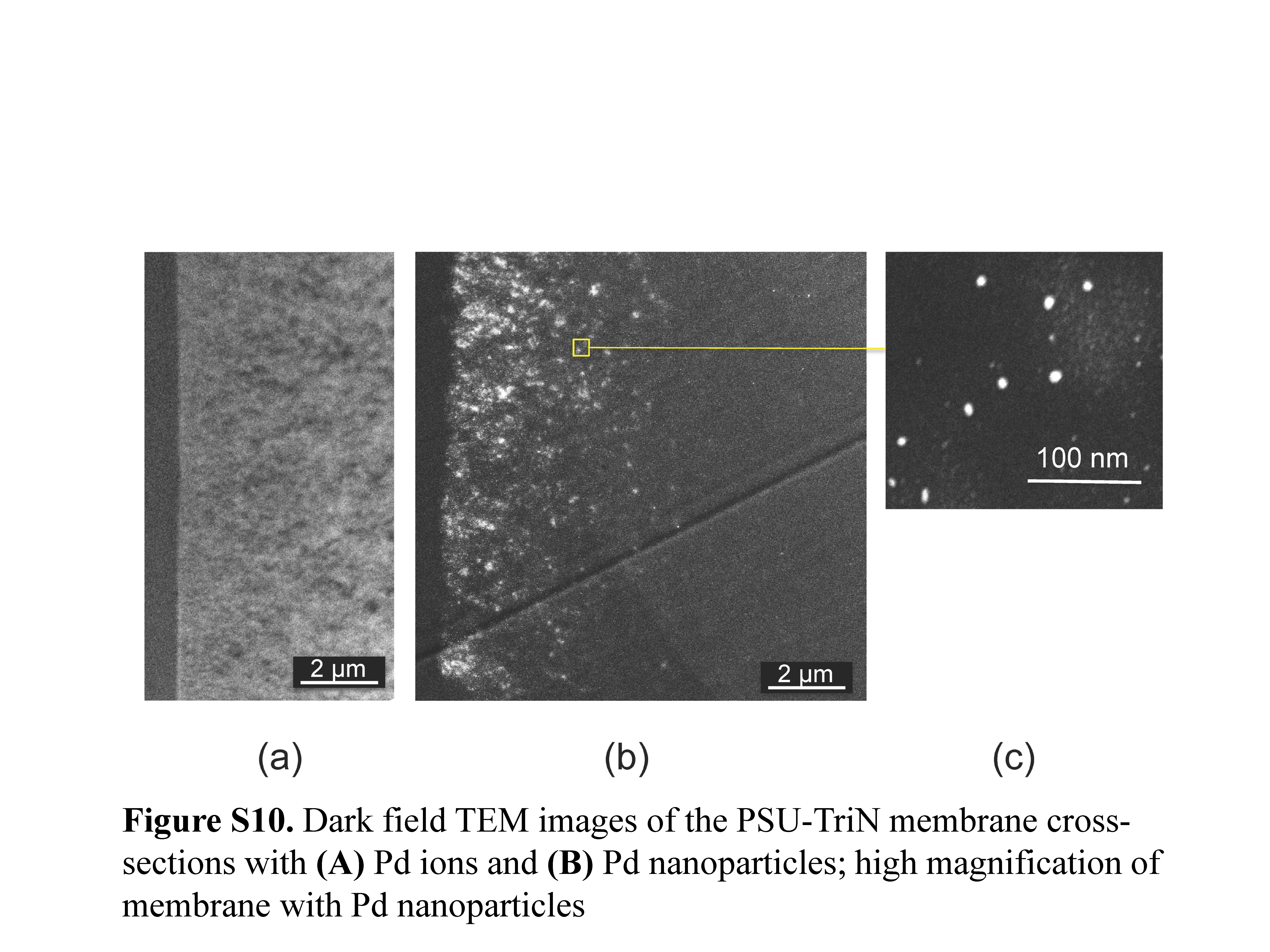


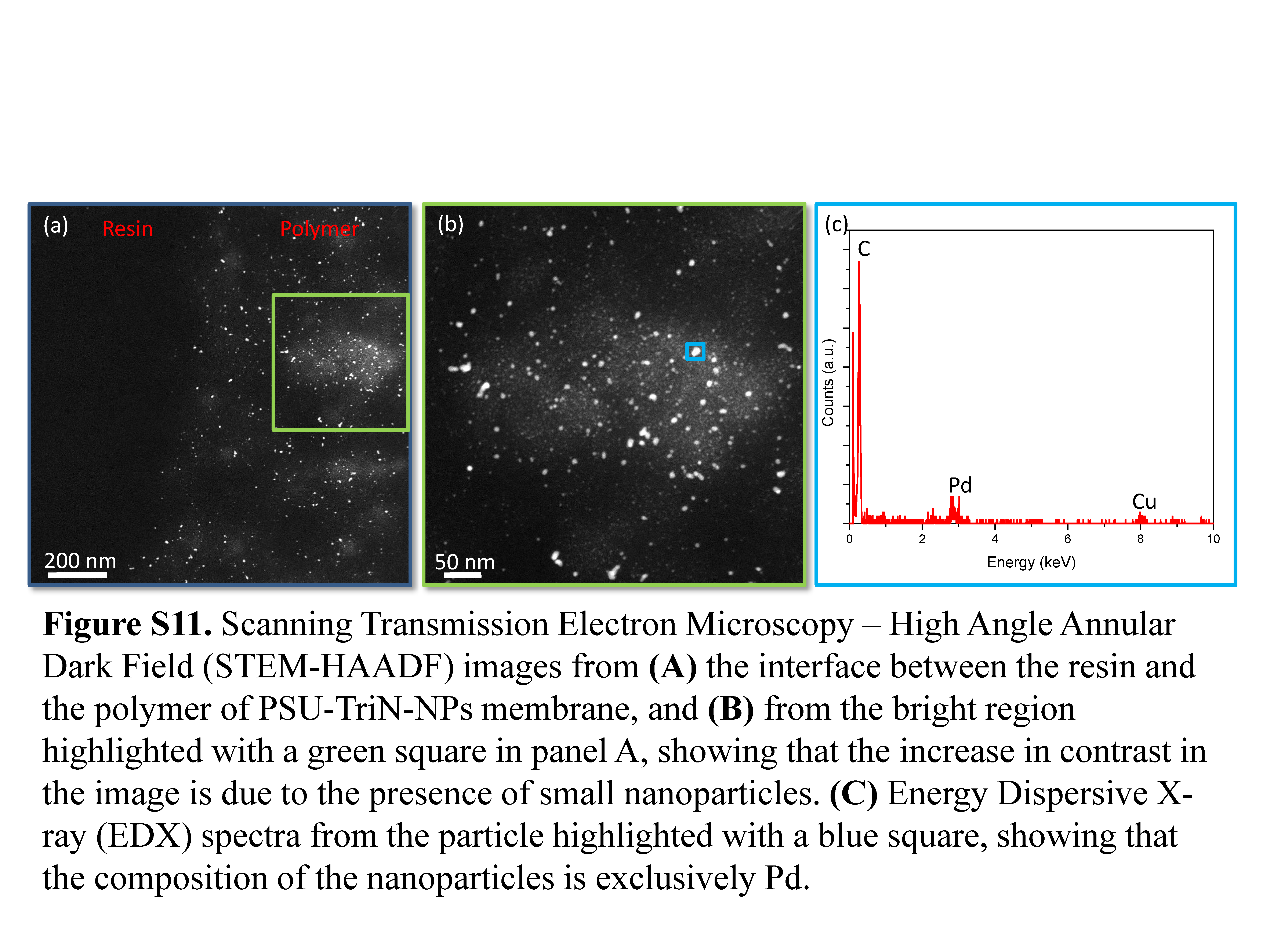

Supplement: Supplementary Information [file srep24289-s1.doc]
